# The Protective Effects and Potential Mechanisms of *Ligusticum chuanxiong*: Focus on Anti-Inflammatory, Antioxidant, and Antiapoptotic Activities

**DOI:** 10.1155/2020/8205983

**Published:** 2020-10-19

**Authors:** Jinhui Shi, Ruiyue Li, Siyu Yang, Yeelin Phang, Changwu Zheng, Hongmei Zhang

**Affiliations:** School of Pharmacy, Shanghai University of Traditional Chinese Medicine, Shanghai 201203, China

## Abstract

*Ligusticum chuanxiong* (LC) is a Chinese materia medica which is widely used in clinical settings to treat headaches, blood extravasation, and arthritis. Recent studies demonstrate that LC possesses versatile pharmacological functions, including antiatherosclerosis, antimigraine, antiaging, and anticancer properties. Moreover, LC also shows protective effects in the progression of different diseases that damage somatic cells. Oxidative stress and inflammation, which can induce somatic cell apoptosis, are the main factors associated with an abundance of diseases, whose progresses can be reversed by LC. In order to comprehensively review the molecular mechanisms associated with the protective effects of LC, we collected and integrated all its related studies on the anti-inflammatory, antioxidant, and antiapoptotic effects. The results show that LC could exhibit the mentioned biological activities by modulating several signaling pathways, specifically the NF-*κ*B, Nrf2, protein kinase, and caspase-3 pathways. In future investigations, the pharmacokinetic properties of bioactive compounds in LC and the signaling pathway modulation of LC could be focused.

## 1. Introduction

Emerging evidence demonstrates that oxidative stress and inflammation are two predominant factors inducing severe organ damage which could result in digestive diseases, cardiovascular diseases, and diabetes [1, 2]. Generally, the basal level of ROS is essential for cell survival and growth since it plays a crucial role in the modulation of several signaling pathways [3]. For instance, the phosphatidylinositol 3-kinases/protein kinase B (PI3K/Akt) pathway is one of the best-known ROS-regulated pathways controlling cellular proliferation. While a low level of ROS is important for several physiological processes [4], its overproduction by peroxisome and mitochondria could induce oxidative stress in the human body [5, 6]. This in turn can lead to cell apoptosis by damaging its organelle and chromatin [7] as well as trigger immune response and inflammation due to the degeneration of biomacromolecules such as DNA and proteins [8]. The enhanced inflammatory response due to oxidative stress is closely associated with increased risk of cardiovascular diseases such as atherosclerosis and hypertension [9]. Therefore, scientists have taken a significant step forward to search for suitable candidates that can protect against oxidative stress and inflammation.

As natural products are found to be imperative sources of medicinal agents that exhibit anti-inflammatory and antioxidant activities [10, 11], a diverse group of Chinese materia medica have also generated a great deal of interest [12–14]. According to traditional Chinese medicine theories and recent studies, *Ligusticum chuanxiong* (LC) possesses a protective effect which ameliorates the lesions of various diseases induced by inflammation, oxidative stress, and apoptosis [15–17]. It is a deciduous plant belonging to the Umbelliferae family and is predominantly distributed in the Sichuan Province in southwest China. Most of the effective components of this traditional herb come from phenolic acids, phthalide lactones, polysaccharides, steroids, volatile oils, and alkaloids [16, 18–20]. Traditionally, LC is often used as medicine to treat headache, blood extravasation, and arthritis [21]. A recent study by Liu and coworkers reported that LC shows protective activity against myocardial ischemia [22] while another paper published by Wang suggested that LC could reduce isoproterenol-induced myocardial ischemia injury in rat models [23]. In addition, LC is also found to possess antineoplastic potential on HeLa cells [24]. Its protective effect on D-galactose-induced liver and kidney injury is potentially linked with alleviation of oxidative stress and inflammatory response [25]. These functions proposed the possible use of LC as a treatment for a range of oxidative stress- and inflammation-associated diseases which include but not limited to atherosclerosis, migraine, and hepatic fibrosis [26–28].

Herein, this review aims to highlight the protective effects of LC and its associated molecular mechanisms with a special focus on its anti-inflammatory, antioxidant, and antiapoptotic effects. The references and articles presented have been collected and analyzed through several online databases, including ScienceDirect, China National Knowledge Infrastructure (CNKI), Springer, and Wiley.

## 2. Molecular Mechanisms of LC's Anti-Inflammatory, Antioxidant, and Antiapoptotic Effects

**Figure 1:**
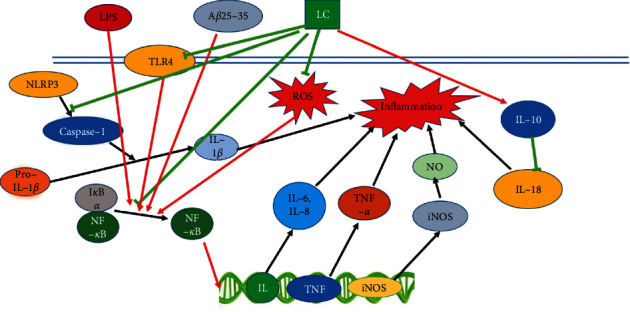
The molecular mechanism involves in the protective effects of LC.

**Figure 2:**
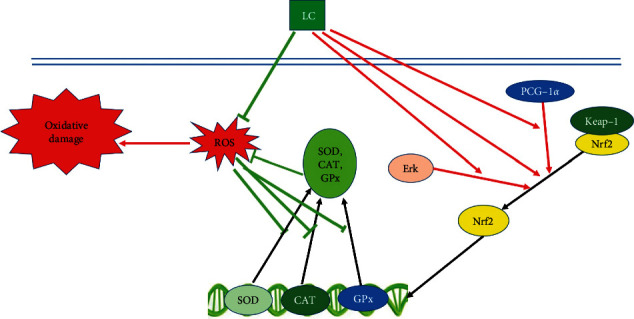
The antioxidant mechanism of LC.

**Figure 3:**
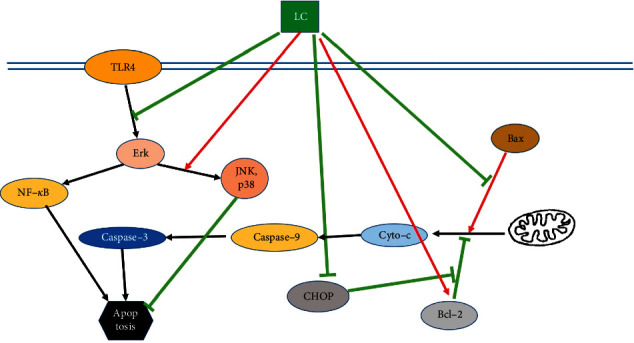
The antiapoptotic mechanism of LC.

Over the past decades, tremendous efforts have been made in unraveling the protective effect and the molecular mechanisms of LC in diseases that are closely linked to oxidative stress and inflammation. In this section, the anti-inflammatory, antioxidant, and antiapoptotic properties of LC are discussed. The associated molecular mechanisms and signaling pathways are studied in depth as well (Table 1), and the corresponding schemes are summarized in Figures 1–3.

### 2.1. Anti-Inflammatory Actions

Inflammation is a complex biological response of the immune system that is triggered by harmful endogenous and/or exogenous stimuli. While inflammation serves as a vital survival mechanism to fight against illness or injury, systemic chronic inflammation has been shown to be associated with increased risk of developing a variety of common chronic diseases [42] such as Alzheimer's disease [43] and diabetic nephropathy [44, 45]. The inflammation-induced somatic cell apoptosis might result in severe lesions in organs, promoting the disease progression. Following the discovery that LC exhibits anti-inflammatory action, the molecular mechanisms and signaling pathways of how LC modulates inflammatory response becomes a high-value target for the discovery of new leads that can slow down such disease progression.

In recent studies, LC has been shown to possess anti-inflammatory properties *via* suppression of proinflammatory cytokines [25, 29]. Usually, lipopolysaccharides (LPS) act as an endotoxin to induce the production of many proinflammatory cytokines in both *in vitro* and *in vivo* assays to study the anti-inflammatory properties of an active component. Several studies reported that LC plays a role in the suppression of LPS-induced nitric oxide (NO), tumor necrosis factor-*α* (TNF-*α*), and interleukin-1*β* (IL-1*β*) production in RAW 264.7 cells and BV-2 cells [15, 30, 31, 46]. Or et al. also suggested that LC prohibits the activation of BV-2 cells induced by LPS. Upon further investigations, they discovered that senkyunolide A and ligustilide are the active anti-inflammatory components in LC, and these two lactones are able to suppress the secretion of TNF-*α* directly and reduce inducible nitric oxide synthase (iNOS) activity [15]. Meanwhile, another study by Liu et al. showed that LC inhibits degeneration of inhibitor *κ*B-*α* (I*κ*B*α*) by blocking the NF-*κ*B/p38 pathway and suppressing the expression of several proinflammatory cytokines in RAW 264.7 cells [32].

In addition to proinflammatory cytokines, a high level of reactive oxygen species (ROS) is also noxious for living organisms because it might damage proteins and lipids and also activate the NF-*κ*B pathway which might lead to inflammation. In a study by Mahmoud et al., ferulic acid has been found to block the NF-*κ*B pathway which is activated by ROS [47]. Moreover, Hu et al. proposed that senkyunolide I, another bioactive compound in LC, is able to reduce the ROS-induced production of TNF-*α*, IL-1*β*, IL-6, and IFN-*γ* in BV-2 cells [29].

In 2015, Wu et al. studied the anti-inflammatory responses that LC has on platelet-derived growth factor- (PDGF-) induced hepatic stellate cells from a CCl_4_-treated rat model. They found that ligustrazine efficiently suppresses the production of caspase-1 which is induced by nucleotide-binding oligomerization domain-like receptor 3 (NLRP3) in hepatic stellate cells, as well as reduces the levels of IL-1*β* and IL-18 through inhibition of the NLRP3/NF-*κ*B pathway [48].

Moreover, the protein kinase/NF-*κ*B is another pathway which might induce inflammation. Lei et al. found that in the ApoE^−/−^ mouse model, phthalides in LC inhibit the expression of Akt and via downregulation of the NF-*κ*B signaling pathway, and ligustilide is able to suppress the expression of activator protein-1 (AP-1) which is a promoter of CD137. Through these two pathways, secretion of many proinflammatory cytokines can be suppressed [28].

Besides, Kim et al. reported that ligustrazine, an alkaloid in LC, significantly inhibits the production of TNF-*α*, IL-1*β*, and monocyte chemoattractant protein-1 (MCP-1) in microglial cells which are activated by A*β*_25-35_, a stimulus in the brain that can activate inflammatory response in microglial cells, through the interdiction of the NF-*κ*B pathway [33].

In the development of intracerebral hemorrhages, the expression of Toll-like receptor 4 (TLR4) is enhanced, and this could lead to an increase in the levels of TNF-*α*, IL-6, and IL-1*β* through the TLR4/NF-*κ*B pathway, followed by aggravation of brain lesions. Fortunately, recent investigations have revealed that the use of LC could significantly suppress the level of proinflammatory cytokines induced by TLR4 [49, 50].

In addition, the study published by Hu et al. reported ligustrazine (200 mg/kg) could reduce inflammation in a rat model with spinal cord injury by stimulating the secretion of IL-10 that is able to neutralize IL-18, a proinflammatory cytokine [35].

The studies mentioned above demonstrated that LC could inhibit the release of various proinflammatory cytokines which is activated by different stimuli in diverse cell lines, providing evidence that LC possesses anti-inflammatory action.

### 2.2. Antioxidant Actions

Oxidative stress is one of the main mechanisms that leads to damage of cell structure and inducement of many maladies. Inside cells, low or moderate concentration of ROS is necessary for cell functioning, but high concentration might result in oxidative stress and damage of biomacromolecules and finally leads to cell apoptosis and organ lesion [3]. Therefore, the modulation of ROS helps to decrease oxidative stress and limit cell damage [51, 52]. Recent studies have shown that LC possesses excellent ROS scavenging ability and has the potential to stimulate the expression of antioxidant enzymes such as superoxide dismutase (SOD), catalase (CAT), and glutathione peroxidase (GPx) [34, 53, 54]. In this section, we reviewed recent studies on the antioxidant potentials of LC and its underlying mechanisms.

According to recent studies, polysaccharides in LC are able to scavenge ROS in an *in vitro* model. As polysaccharides are strong reducers, oxidative stress induced by diverse oxidative reagents, including 1,1-diphenyl-2-picrylhydrazyl (DPPH), H_2_O_2_, and 2′-azinobis-(3-ethylbenzothiazoline-6-sulfonic acid), could be attenuated by them in LC [16, 34, 53]. Among the three different polysaccharides in LC isolated by Hu et al., two of them show a high to moderate DPPH scavenging effect with IC_50_ values at 2.95 mg/mL and 8 mg/mL, respectively [16]. Similarly, Liu et al. also isolated polysaccharides from LC and assayed their antioxidant abilities. In their experiment, polysaccharides in LC can dose-dependently (0.05–1.5 mg/mL) scavenge diverse oxidative reagents. Notably, when H_2_O_2_ is used as the oxidative reagent, the scavenging ability of these polysaccharides is close to butylated hydroxytoluene, which is a compound with a strong reducing capacity [53]. However, as these experiments are carried out *in vitro*, the membrane permeability of these polysaccharides remains unclear, and this could be a perspective for future research. Moreover, ferulic acid can also scavenge ROS because it possesses the bulky conjugation system with a side chain [36, 37], which might be another component that contributes to the ROS scavenging ability of LC.

The modulation of antioxidant enzymes is another common pathway responsible for the antioxidant properties of LC. Under oxidative stress, the activity of antioxidant enzymes usually declines and results in an increase in ROS [38]. Recent studies demonstrate that LC can promote the expression of antioxidant enzymes, including SOD, CAT, and GPx, in somatic cells [25, 40, 54]. Through the nuclear factor erythroid 2-related factor 2 (Nrf2) pathway, organisms are able to protect against oxidative stress. In a study by Wang et al., water or ethanol extract of LC (600 mg/kg and 1200 mg/kg) can ameliorate the oxidative stress in the isoproterenol-treated rat model. They further analyzed the variations of SOD, Nrf2, and HO-1 in cardiac muscle tissue, and the results show that the levels of these proteins increased in the LC-treated group. This suggests that LC can activate the Nrf2 pathway to alleviate oxidative stress. Notably, the level of SOD is higher in the ethanol extract group, which implies that the main component responsible for the activation of the Nrf2 pathway could be phthalide lactones [41]. Moreover, according to a study by Gong et al., ferulic acid can increase the progress of Nrf2 which segregates from Keap-1 and combines with the promoter ARE in the cell nucleus to enhance the expression of heme oxygenase-1 (HO-1) in oxidation-sensitive dye 2′,7′-dichlorofluorescein diacetate-treated H9C2 cells [36]. In addition, peroxisome proliferator-activated receptor-gamma coactivator-1*α* (PGC-1*α*) is another upstream signal of the Nrf2 pathway. Huang et al. found that, through the PCG-1*α*/Nrf2 pathway, polysaccharides (10–50 mg/kg) in LC upregulate the levels of antioxidants SOD1, SOD2, CAT, glutathione S-transferase P-1, and glutathione S-transferase M-1, which all play roles as antioxidants [34]. Meanwhile, LC is found to increase the phosphorylation of Erk1/2 which could ultimately increase the phosphorylation of Nrf2 [39].

### 2.3. Antiapoptotic Actions

Oxidative stress and high level of inflammatory cytokines are linked to an increase in cell apoptosis, which could lead to various organ injuries. Two pathways to control cell apoptosis include suppression of inflammation and oxidative stress, as well as modulation of apoptotic factors [11, 32, 55].

Recent studies proposed that LC shows antiapoptotic actions by modulating protein kinase pathways. In an investigation from Lin et al., the butanol part of ethanol-extracted LC (1–100 *μ*M) can increase the viability of 3-(4,5-dimethylthiazol-2-yl)-2,5-diphenyl tetrazolium bromide- (MTT-) treated PC12 cells, but this function diminishes at a concentration of 300 *μ*M [56]. The mechanism is that LC increases the level of cAMP response element-binding protein (CREB) which possesses antiapoptotic effect through the protein kinase A (PKA) pathway [56]. In their further investigations, they found that LC can enhance the level of Erk, another downstream signal of PKA that is responsible for inhibition of apoptosis. They also found that LC can increase the level of phosphorylated c-Jun NH_2_-terminal kinase (JNK) and phosphorylated p38 protein belonging to the MAPK family which possesses antiapoptotic ability [57]. However, in another study conducted by Gu et al., Erk acts as a proapoptosis factor in the TLR4/NK-*κ*B pathway. In this experiment, water extraction of LC is able to reduce the content of cerebral water in MACO rat model owing to the antiapoptotic action of LC through inhibition of TLR4-activated Erk [49].

Caspase family is another category of proteins related to apoptosis. Gu et al. reported that water-extracted LC inhibits the production of caspase-3 and caspase-12 in the MCAO rat model [49]. Furthermore, another experiment conducted by Gu et al. demonstrates that total phenolic acids in LC (0.2 mg/mL) could downregulate Bax/Bcl-2 which can cause the activation of caspase-3 in MTT-treated human umbilical vein endothelial cells [58]. LC could also inhibit the activation of caspase-3 directly, increasing the viability of MTT-treated PC12 cells [59].

The modulations of other proteins which are crucial for apoptosis are also researched. In an experiment conducted by Wang et al., ethanol-extracted LC could significantly increase the level of growth-associated protein-43 (GAP-43) which is related to the growth of hippocampal in a microsphere-induced cerebral embolism rat model. As a result, the recovery of neurons significantly increases [60]. Another study found that unfolded protein in the endoplasmic reticulum is increased in an MCAO rat model, which in turn induces endoplasmic reticulum stress and enhances the level of CHOP protein which can inhibit the function of Bcl-2 and result in apoptosis of neurons. Nevertheless, this increase in CHOP protein can be suppressed by water-extracted LC [61].

Through the modulations of caspases, the Bcl-2 family proteins, and other related protein kinases, LC can play a significant role in the protection of cells in diseases which involve drastic somatic cell apoptosis such as stroke and neurodegenerative diseases.

## 3. Protective Effect of LC

With its diverse anti-inflammatory, antioxidant, and antiapoptotic properties, LC demonstrates protective effect in various diseases (Table 2).

Through anti-inflammatory action, LC can protect an organism from multiple lesions. Atherosclerosis is shown to have a strong correlation with oxidized lipid deposition, and it has been suggested to induce the production of proinflammatory cytokines such as TNF-*α*. Through the NF-*κ*B pathway, the levels of intercellular cell adhesion molecule-1 and vascular cell adhesion molecule-1 increase in human umbilical vein endothelial cells and result in the formation of plaques [28, 62]. In a study by Xiao et al., total lactones in LC (30 and 60 mg/kg, 3 months) can suppress the serum lipid level in an ApoE^−/−^ rat model with high-fat diet, suggesting that LC has a potential on the medical treatment of atherosclerosis. They further found that LC can dose-dependently (3.125–25 *μ*g/mL) inhibit the levels of intercellular adhesion molecule-1 and vascular cellular adhesion molecule-1 via inhibition of the NF-*κ*B pathway [62]. Furthermore, Lei et al. reported that the treatment of atherosclerosis with phthalides in LC in the ApoE^−/−^ mouse model is related to the inhibition of NF-*κ*B and AP-1 expressions and as well as suppression of CD137 level which is considered as a biomarker of atherosclerosis [28].

Furthermore, Han et al. found that phthalides in LC play a protective role against damage associated with intracerebral hemorrhaging. Through the inhibition of the TLR4/NF-*κ*B pathway, the secretion of TNF-*α*, IL-6, and IL-1*β* decreases, and the injury to the brain due to intracerebral hemorrhage can be attenuated [50, 51]. In the progression of cerebral trauma, the permeability of the blood-brain barrier decreases due to the increase of TNF-*α* and IL-1*β*. Wang et al. reported that ligustrazine (20 mg/kg) can decrease the level of TNF-*α* and IL-1*β* in the plasma of a rat model [63]. Additionally, through the NOS inhibition and the monoamine neurotransmitter modulation, senkyunolide I (16 and 32 mg/kg) showed treatment potential for migraine rats [64].

LC also shows protective effects on other organs during the inflammatory progress. Diabetes induces injury to the kidney by stimulating inflammatory factors. Yang et al. reported that ethanol-extracted LC (25, 50, and 100 mg/kg) has a therapeutic effect in streptozotocin-induced diabetic nephropathy mice through the inhibition of the NF-*κ*B pathway, which subsequently induces the production of TNF-*α* [65]. Intervertebral disc degeneration is another disease induced by inflammatory damage on organs. In a study by Wang et al., ligustilide (10 mg/kg) suppresses the level of IL-1*β*, downregulates the expression of cyclooxygenase-2, iNOS, TNF-*α*, and IL-6, and upregulates IL-10 and transforming growth factor-*β* (TGF-*β*), leading to an antiapoptotic function in the nucleus pulposus cells of the intervertebral disc [46].

Oxidative stress often causes injury to diverse organs by damaging biomacromolecules, which leads to apoptosis of somatic cells. Sue et al. reported that ligustrazine (80 mg/kg) can induce the expression of HO-1 in gentamicin-treated mice by which oxidative damage on renal can be ameliorated [66]. The central nervous system can also be affected by oxidative stress. For instance, Hu et al. found that senkyunolide I (36 and 72 mg/kg) can alleviate damage to the cerebrum caused by oxidative stress in ischemic stroke. In this progress, LC can increase the level of HO-1 and SOD through activation of the p-Erk/Nrf2 pathway [39]. In addition, ligustilide has been shown to decrease D-galactose-induced ROS production which could activate caspase-3 and cause neurotoxicity that may result in neuron apoptosis and cognitive impairment, respectively [67].

This evidence demonstrates that LC possesses protective effect in the progress of various diseases induced by an inflammatory response and oxidative damage. However, there are also many other diseases that can be alleviated by the protective effect of LC, but the mechanisms are still unclear. For instance, Zou et al. reported that LC can be used to treat hypertension [68] and Liu et al. reported the medical effect of LC on myocardial ischemia [22]. It is valuable to explore their underlying mechanisms in the future.

## 4. Conclusion and Future Perspectives

As a Chinese materia medica, LC is used clinically for various diseases, including migraine, rheumatism, and amenorrhea. Pharmacological investigations demonstrate that LC possesses antiatherosclerosis, anticancer, antioxidant, antiaging, and antihypertensive properties. Furthermore, molecular mechanistic studies show that LC can modulate a diverse array of cytokines, which allows LC to possess anti-inflammatory, antioxidant, and antiapoptotic effects. Through the blocking of the NF-*κ*B pathway and upstream signals, LC can suppress the expression of proinflammatory cytokines, by which the progression of inflammation could be inhibited. The levels of various antioxidant enzymes could also be enhanced via activation of the Nrf2 pathway. LC also possesses strong scavenging ability that directly interacts with ROS. Additionally, through the modulation of caspase-3, Bax family, and MAPK family and their upstream signals in different cell types, LC controls apoptosis. Also, one pathway might coordinate or neutralize another pathway. For example, ROS activates the NF-*κ*B pathway which might result in inflammation, while scavenging ROS can ameliorate oxidative stress and inflammation. Moreover, LC can activate Erk under a serum-deprived environment and show antiapoptotic function, and it can also prohibit TLR4-induced Erk in MCAO rats. Interestingly, the experimental model of the former study is PC12 cells, which is a category of neuron cells. It is valuable to ascertain the interaction between LC and Erk protein in the neuron in future investigations. Generally, via the three pathways discussed, LC shows protective effects as an anti-inflammatory, an antioxidant, and a modulator of apoptosis during the progression of various diseases.

However, there are still research gaps to be fulfilled in order to comprehensively understand the mechanism of the protective functions of LC. We propose the following two suggestions for future investigations of LC:

1. Most of the mechanisms mentioned above occurred intracellularly, and the pathways of how LC enters into cells are important for the understanding of the interaction of LC with the plasma membrane.
2. Via the synergism of antioxidant, anti-inflammatory, and antiapoptotic effects, LC showed medical treatment's potential to ischemic stroke; therefore, in order to validate the bioactive compounds in LC as drug, further study on its blood-brain barrier permeability and pharmacokinetic properties should be carried out.

## Figures and Tables

**Table 1:** Protective effects of LC.

Drug	Dose	Pathway	Model	Ref
*Anti-inflammatory*				
Ligustrazine	50, 100, 200 mg/kg	NLRP3/NF-*κ*B↓	CCl_4_-treated rats	[29]
Ligustilide	—	Protein kinase/NF-*κ*B↓	ApoE^−/−^ mice	[15]
Ligustrazine	—	NF-*κ*B↓	A*β*_25-35_-treated microglial cells	[30]
Water extraction	—	TLR4/NF-*κ*B↓	MACO rat	[31]
Ligustrazine	200 mg/kg	NF-*κ*B↓, IL-10↓	Spinal cord injury rats	[32]

*Antioxidant*				
Polysaccharides	2.95 mg/mL, 8 mg/mL	—	DPPH	[11]
Polysaccharides	0.05–1.5 mg/mL	—	DPPH and H_2_O_2_	[33]
Ethanol extraction	600, 1200 mg/kg	Nrf2↑	Isoproterenol-treated rats	[34]
Water extraction	600, 1200 mg/kg	Nrf2↑	Isoproterenol-treated rats	[34]
Ferulic acid	—	Keap/Nrf2/HO-1↑	2′,7′-Dichlorofluorescein diacetate-treated H9C2 cells	[35]
Polysaccharides	10–50 mg/kg	PCG-1*α*/Nrf2↑	Rat model	[28]

*Antiapoptosis*				
Butanol part of ethanol extraction	1–100 *μ*M	PKA/CREB↑	MTT-treated PC12 cells	[36]
Butanol part of ethanol extraction	1–100 *μ*M	Erk↑	MTT-treated PC12 cells	[37]
Water extraction	—	TLR4↓	MACO rats	[38]
Water extraction	—	Caspase-3↓	MACO rats	[38]
Total phenolic acid	0.2 mg/mL	Bax/Bcl-2↓	MTT-treated human umbilical vein endothelial cells	[39]
Ethanol extraction	—	GAP-43↑	Microsphere-induced cerebral embolism rats	[40]
Water extraction	—	CHP proteins↓	MACO rats	[41]

**Table 2:** Diseases ameliorated by LC.

Disease	Drug	Dosage (mg/kg)	Model	Ref
Atherosclerosis	Total lactone	30, 60	ApoE^−/−^ mice	[56]
Cerebral trauma	Ligustrazine	20	Controlled cortical impact mice	[49]
Migraine	Senkyunolide I	16, 32	Nitroglycerin-treated rats	[58]
Diabetic nephropathy	Ethanol extraction	25, 50, 100	Streptozotocin-treated mice	[24]
Intervertebral disc degeneration	Ligustilide	10	Mice treated by surgery	[42]
Oxidative damage on renal	Ligustrazine	80	Gentamicin-treated mice	[59]
Ischemic cerebral stroke	Senkyunolide I	36, 72	Ischemia-reperfusion injured rats	[53]
Cognitive impairment	Ligustilide	—	D-galactose-treated mice	[60]

## Abbreviation

A*β*: Amyloid-*β* peptide
Akt: Protein kinase B
AP-1: Activator protein 1
CAT: Catalase
CNKI: China National Knowledge Infrastructure
CREB: cAMP response element-binding protein
DPPH: 1,1-Diphenyl-2-picrylhydrazyl
Erk1/2: Extracellular signal-regulated kinase 1/2
GAP-43: Growth-associated protein-43
GPx: Glutathione peroxidase
HO-1: Heme oxygenase 1
HSC: Hepatic stellate cells
I*κ*B*α*: Inhibitor kappa B alpha
IKK: Inhibitor of NF-*κ*B kinase
IL: Interleukin
iNOS: Inducible nitric oxide synthase
JNK: c-Jun NH2-terminal kinase
LC: Ligusticum chuanxiong
LPS: Lipopolysaccharides
MAPK: Mitogen-activated protein kinase
MCAO: Middle cerebral artery occlusion
MCP-1: Monotype chemoattractant protein-1
MTT: 3-(4,5-Dimethylthiazol-2-yl)-2,5-diphenyl tetrazolium bromide
MyD88: Myeloid differentiation factor 88
NF-*κ*B: Nuclear factor-kappa B
NLRP3: Nucleotide-binding oligomerization domain-like receptor 3
NO: Nitric oxide
Nrf2: Nuclear factor erythroid 2-related factor 2
PC12: Pheochromocytoma 12
PDGF: Platelet-derived growth factor
PI3K: phosphatidylinositol 3-kinase
p-Erk: Phosphorylated Erk
PGC-1*α*: Peroxisome proliferator-activated receptor-gamma coactivator 1*α*
PKA: Protein kinase A
ROS: Reactive oxygen species
SOD: Superoxide dismutase
TGF-*β*: Transforming growth factor-*β*
TLR4: Toll-like receptor 4
TKA1: Tyrosine kinase activator protein 1
TNF-*α*: Tumor necrosis factor-alpha
TRAF6: TNF receptor-associated factor 6.
